# Alteration of chromatin states perturb the transcription regulation of gene during hydronephrosis

**DOI:** 10.3389/fgene.2025.1396073

**Published:** 2025-02-17

**Authors:** Xiao-Hui Wang, Shu-Feng Zhang, Hai-Ying Wu, Jian Gao, Lin Wang, Yao Yin, Xuhui Wang

**Affiliations:** ^1^ Department of Pediatric Surgery, Henan Provincial People’s Hospital, Zhengzhou, China; ^2^ Department of Obstetrics, Henan Provincial People’s Hospital, Zhengzhou, China; ^3^ Department of Medical Affairs, Henan Provincial People’s Hospital, Zhengzhou, China

**Keywords:** hydronephrosis, atac seq, BS-seq, GO term and KEGG pathways, OTUD6B

## Abstract

**Background:**

Gene expression is abnormal in disease compared to normal tissue same as the regulatory elements. Regulatory element binding with transcription factors managed transcription of gene, which usually require chromatin accessible.

**Methods:**

To reveal potential epigenetic mechanism during hydronephrosis, we first used RNA-seq to finger out the disfunction genes during hydronephrosis, then combined with ATAC-seq, and BS-seq to reveal the related disfunction regulatory elements.

**Results:**

Finally, we find that 860 differentially genes and 2429 dynamic chromatin open regions between normal and hydronephrosis tissue. Though, most of disfunction genes and regulatory elements significantly enriched in chronic kidney disease GO term, only small part of regulatory element target genes overlapped with truly disfunction genes. And we also find out an important gene OTUD6B, which overexpression in disease tissue is manipulated by distal regulatory element through chromatin loop, and confirm the importance of epigenetic mechanism in disease.

**Conclusion:**

In summary, we found many hub genes and potential therapeutic target during hydronephrosis, and also confirmed that epigenetic play important role in gene expression and relevant in disease progress.

## Background

Hydronephrosis, accompany with ureteral stricture, is a common disease may happen in any age. Usually, the urinary tract infection, obstruction of urinary tract or vesicoureteral reflux is the main cause. Although mild hydronephrosis is self-cure, long-term hydronephrosis can lead to increased pressure and permanent injury in the kidney, which is easy to cause chronic nephritis even kidney failure.

Numerous cell types such as epithelial cells, endothelial cells, stromal cells and immune cells involved in the development of hydronephrosis. Immune cells and interstitial cells are pivotal part of the composition for kidney function, for example, T cell mediate ureteritis and lead to hydronephrosis under intake of short chain fatty acids (Park et al., 2016). Specialized epithelial is critical for maintaining circulation, and reported that the deletion of Keap1 in epithelium will cause hydronephrosis in mice (Noel et al., 2016). But it remains unclear what is master contributors, and the synergistic interactions between different cell types may critical to sustain kidney function.

Many genes involve in maintaining of normal kidney function, such as *CLMP* and *GFRA3*. Former one plays important role in kidney development, deletion of it will result in severe bilateral hydronephrosis (Rathjen and Jüttner, 2023). Latter is one member of *GDNF* family receptor, that *GDNF* is one kind of secreted molecule and involved in ureter budding (Uetani and Bouchard, 2009). Other transcription factors, for example, *GATA3*, *LIM1*, are also important for kidney structure (Chia et al., 2011), (Boualia et al., 2013). Mutation of *Gata3* in mouse embryos cause hydronephrosis at birth, suggesting Gata3 factor is required for urinary tract mutation (Chia et al., 2011). *FOXF1*, another factor in lung development, was also found that mutation leads to hydronephrosis (Bzdęga et al., 2023). Through, little potential key genes or transcription factors have been explored in hydronephrosis, the underlying genetic mechanism remain further investigate. Recently study showed that, change of chromatin states in regulatory element play crucial part in gene expression, and may lead to severe disease (Mirabella et al., 2016), (Klemm et al., 2019). Nevertheless, we still have limited knowledge about alteration of chromatin states between abnormal and normal tissue during hydronephrosis. Comprehensively understand gene expression and related regulatory network in hydronephrosis will help us recognize pathogenesis and find new therapy target of disease.

**FIGURE 1:**
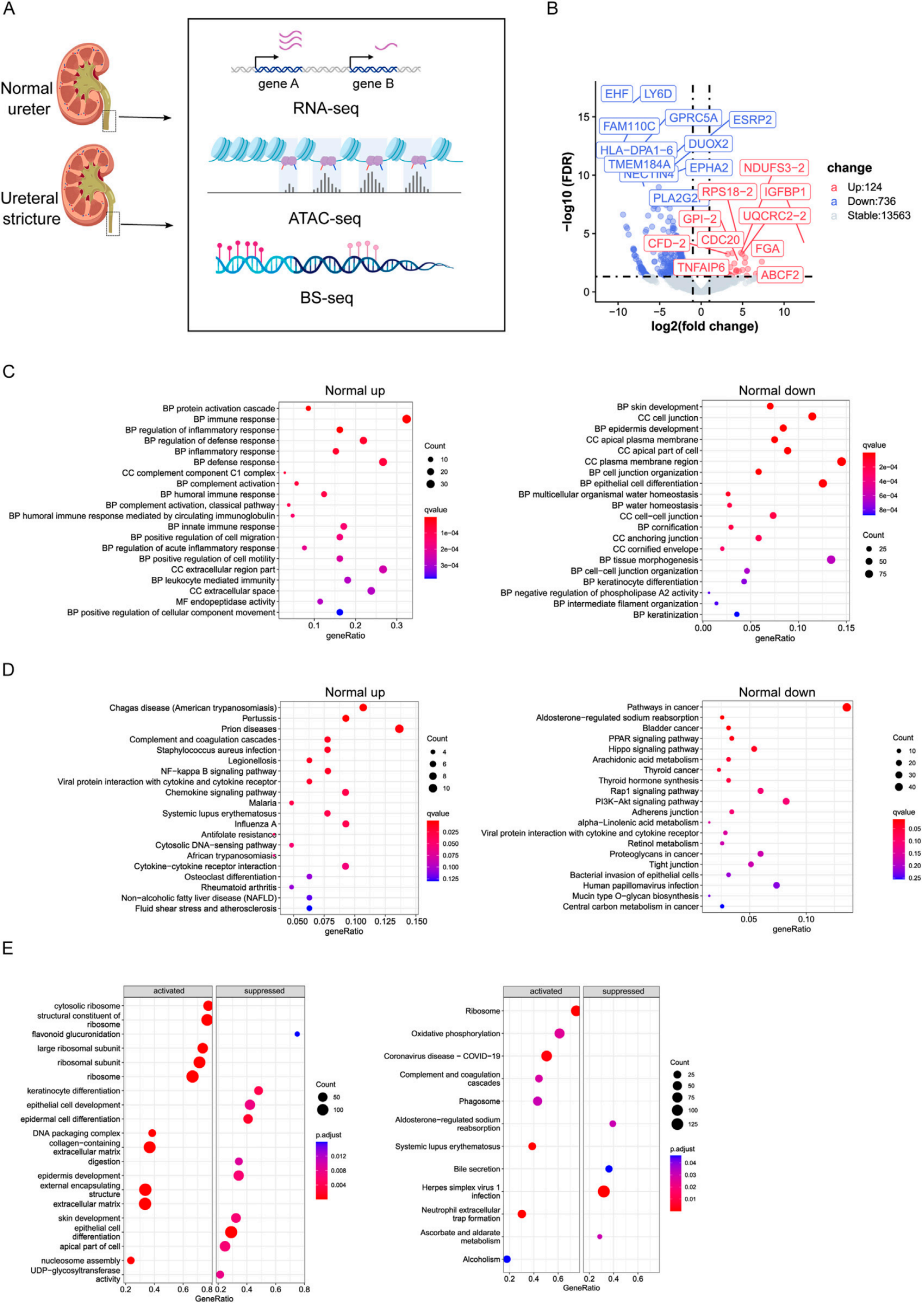
**(A)** Flowchart of experiment. **(B)** Volcano plot of differentially expressed genes between Normal and Ureteral (FDR < 0.05, log fold change > 1). Upregulated genes were higher expressed in Normal. **(C)** GO term enrichment analysis of Normal upregulated genes (left) and Normal downregulated genes (right). **(D)** KEGG enrichment analysis of Normal upregulated genes (left) and Normal downregulated genes (right). **(E)** GSEA GO term analysis (left) and KEGG analysis (right).

We try to detect the differentially express genes (DEGs) between normal and hydronephrosis in this study, then explore the epigenetic change in disease, include the DNA methylation profile and related regulatory element by ATAC-seq detected differentially accessible regions (DARs) (Figure 1A). For visualization of hub-gene in hydronephrosis, we also built protein-protein network (PPI) by STRING. To verify the potential relationship between acquired DEGs and DARs, we further detect the chromatin structure between DEGs and DARs, trying to finger out regulatory mechanism in hydronephrosis.

## Results

### Differentially expressed gene revealed the phospholipase A2 and immune related genes participate in hydronephrosis progression

Differentially expressed genes (DEGs) were detected between normal ureter and ureteral stricture (Figure 1B), and we found 860 DEGs totally (124 upregulated, 736 downregulated, FDR < 0.05, log2FC > 1). Mainly GO enrichment of these DEGs showed that ureteral stricture leads to inflammatory response through humoral immune response and activate defense mechanism (Figure 1C). While downregulated genes related to skin or epithelial cell development or cell junction organization. And the water homeostasis related genes also downregulated. Keratinization or cornification related genes were also change in disease.

Interestingly, Normal downregulated genes enriched at negative regulation of phospholipase A2 (*PLA2*) activity, which have proved to functional in kidney injury (Kim H. et al., 2015), and *PLA2* receptor usually as marker of nephropathy disease diagnose (Figure 1C). Phospholipase A2 group IIF (*PLA2G2F*) usually highly expressed in skin tissue (Prunonosa Cervera et al., 2021). In this study, it expressed more than 200-fold in ureteral stricture compared to normal (24.85333 vs. 0.3733333 FPKM). The activity of *PLA2* positively correlated with concentration, and produce proinflammatory mediators, leads to further development of inflammation then makes the disease worse. In conclusion, Inflammatory of kidney epithelial cells may play important role in hydronephrosis.

KEGG pathway enrichment showed that Normal downregulated genes are enriched in Hippo signaling pathway, PPAR signaling pathway, Rap1 signaling pathway, PI3K-Akt signaling pathway, Bladder cancer (Figure 1D). AREG a ligand of epidermal growth factor receptor (EGFR) and participated in Hippo pathway, was downregulated in our Normal RNA-seq data, confirmed existing report that AREG highly expressed in patients with advanced chronic kidney disease (CKD) and play important role in injury-induced kidney disease (Osakabe et al., 2024). So, KEGG results again emphasized recent study that Hippo pathway may promotes many kidney diseases (Sun et al., 2022), and PPAR signaling pathway play important role in regulation of physiological functions of kidney (Chung et al., 2018). PI3K-Akt involves in lipotoxicity, will regulate nephrin, once decreased will lead to loss of nephrin then effect the apoptosis of podocytes, which observed in adult kidney disease, for example, diabetic nephropathy and HIV-associated nephropathy (Kato et al., 2006), (Ayasolla et al., 2015). KEGG pathway also enriched in Bladder cancer, which is one kind of urinary system diseases. It is interesting that many parasitic diseases, such as chagas disease and malaria enriched in Normal upregulated genes. The potential reason is that these diseases were reported that can damage the kidney and include kidney involvement, and impairment of kidney function in patients is essential to adequately manage and prevent progression to chronic kidney disease and kidney failure (Daher et al., 2022). To sum up, these results demonstrated the overexpressed genes in disease tissue enriched with kidney disease related pathway or GO term.

GSEA analysis corroborates the result from another aspect. GSEA GO enrichment pointed out that Normal repressed genes correlated with epithelial cell differentiation or epidermis development (Figure 1E), while the activated genes in Normal connected to ribosome structure or extracellular matrix. GSEA KEGG showed that activated pathway includes Ribosome, Complement and coagulation cascades, Phagosome, Systemic lupus erythematosus, and Oxidative phosphorylation, which pathway has been proved to activate vascular calcification in kidney disease (Shi et al., 2022). While suppressed pathway contains Bile secretion, Ascorbate and aldarate metabolism. That both bile secretion and ascorbate and aldarate belong to metabolism, which is the main physiological function of kidney.

**FIGURE 2:**
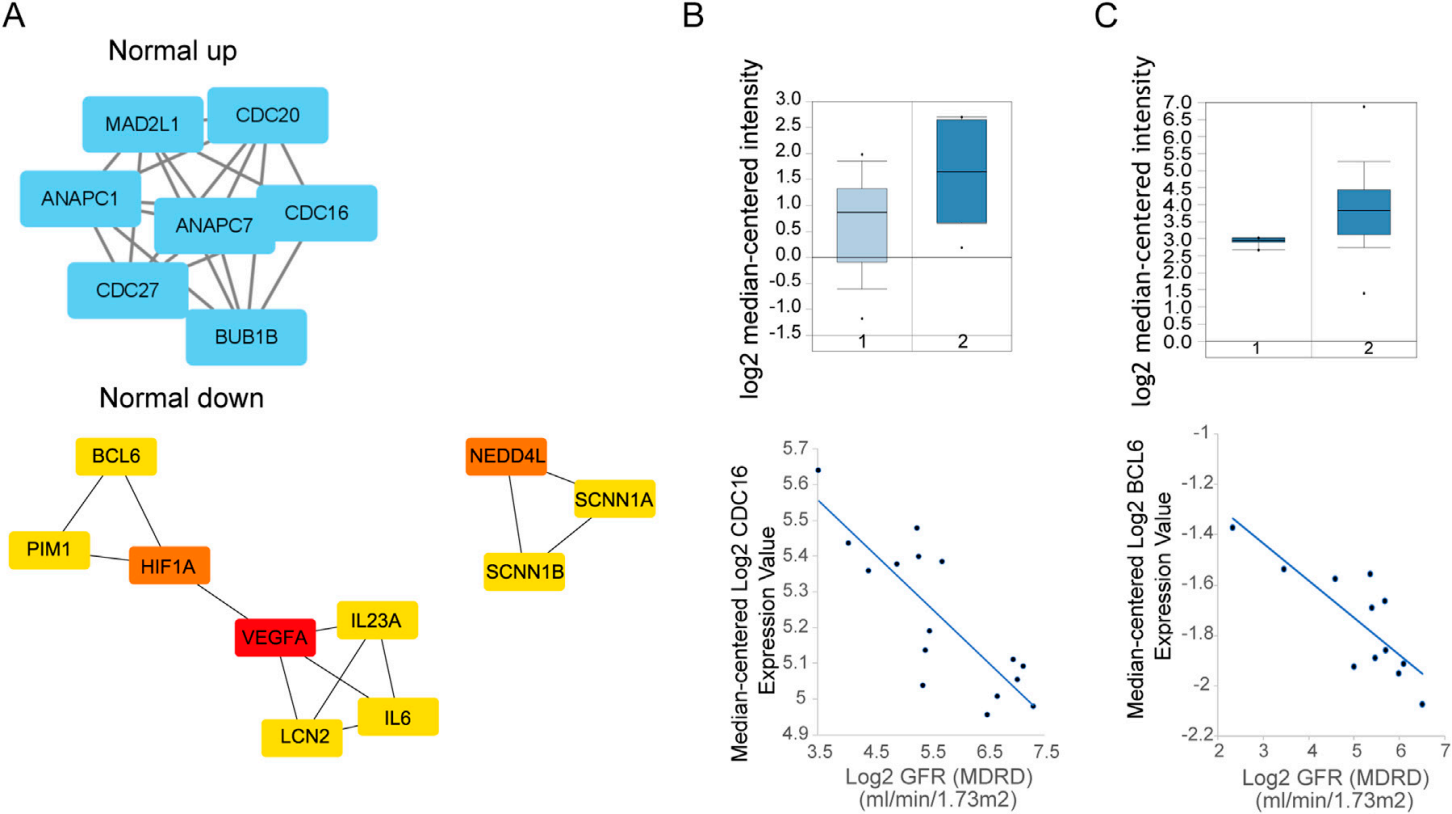
**(A)** PPI network of Normal overexpressed genes (up, left) and downregulated genes up, (right) by STRING. And network of top hub genes identified by MCODE. [**(B)**, Up] Expression of CDC16 between health (1) and disease (2) sample in Nephroseq database. (Down) GFR relationship with expression. [**(C)**, Up] Expression of BCL6 between health (1) and disease (2) sample in Nephroseq database. (Down) GFR relationship with expression.

Protein-protein interaction networks of DEGs were detected by STRING webserver (Szklarczyk et al., 2019), and filtered by high confidence score (0.7) from experiment and database source. Results showed that, the upregulated genes in Normal connected with *CDC16*, *CDC20*, *CXCL8* et al., while the downregulated genes connected with hundreds of genes (Figure 2A). Impressively, the PPI connected repressed genes were enriched in Urinary system disease or skin disease as showed in Table 1.

**TABLE 1:** Disease-gene associations in downregulated gene by STRING PPI network.

Disease	Description	Count in network	Strength	FDR
DOID:2730	Epidermolysis bullosa	7/20	0.99	0.0486
DOID:305	Carcinoma	25/275	0.41	0.0486
DOID:18	Urinary system disease	28/315	0.4	0.0486
DOID:16	Integumentary system disease	41/534	0.33	0.0385

To validate the potential genes is functionally in kidney disease, we choose whole genes (*CDC16*, *CDC20*, *CDC27*, *MAD2L1*, *ANAPC1*, *ANAPC7*, *BUB1B*) from first highest score subnetwork obtained from Cytoscape MCODE plugin from upregulated genes PPI as hub genes. With use of Nephroseq V5 (https://v5.nephroseq.org) (Xu et al., 2021), we compared the genes expression between chronic kidney disease and healthy kidney in clinical sample and get the Glomerular Filtration Rate (GFR) (MDRD) information from this database, analysis showed that the *CDC16* is negatively correlated with GFR from focal segmental glomerulosclerosis samples, which means *CDC16* may promote kidney disease progression (Figure 2B).

The downregulated genes PPI network to cytoscape MCODE showed that second highest score subnetwork have 18 nodes, we choose the first top10 genes as hub genes by plugin cytohubba (Figure 2A). Hub genes include *BCL6*, *PIM1*, *HIF1A*, *VEGFA*, *IL23A*, *IL6*, *LCN2*, *NEDD4L*, *SCNN1A*, *SCNN1B*. Clinical data in Nephroseq V5 also showed that most hub genes upregulated in chronic kidney disease, like *BCL6* (except the *VEGFA*, *NEDD4L*, *SCNN1B*), and negatively correlated with GFR (Figure 2C).

Summarize gene expression from Normal and Ureteral tissue, we found that immune genes highly expressed in disease tissue, and participate in many kidney diseases related pathways and GO. The hub genes from DEGs PPI network confirmed that many critical genes, such as *CDC16* and *BCL6* participate in pathogenesis, and affect the normal function of kidney.

### Chromatin state change related to differentially genes in hydronephrosis

**FIGURE 3:**
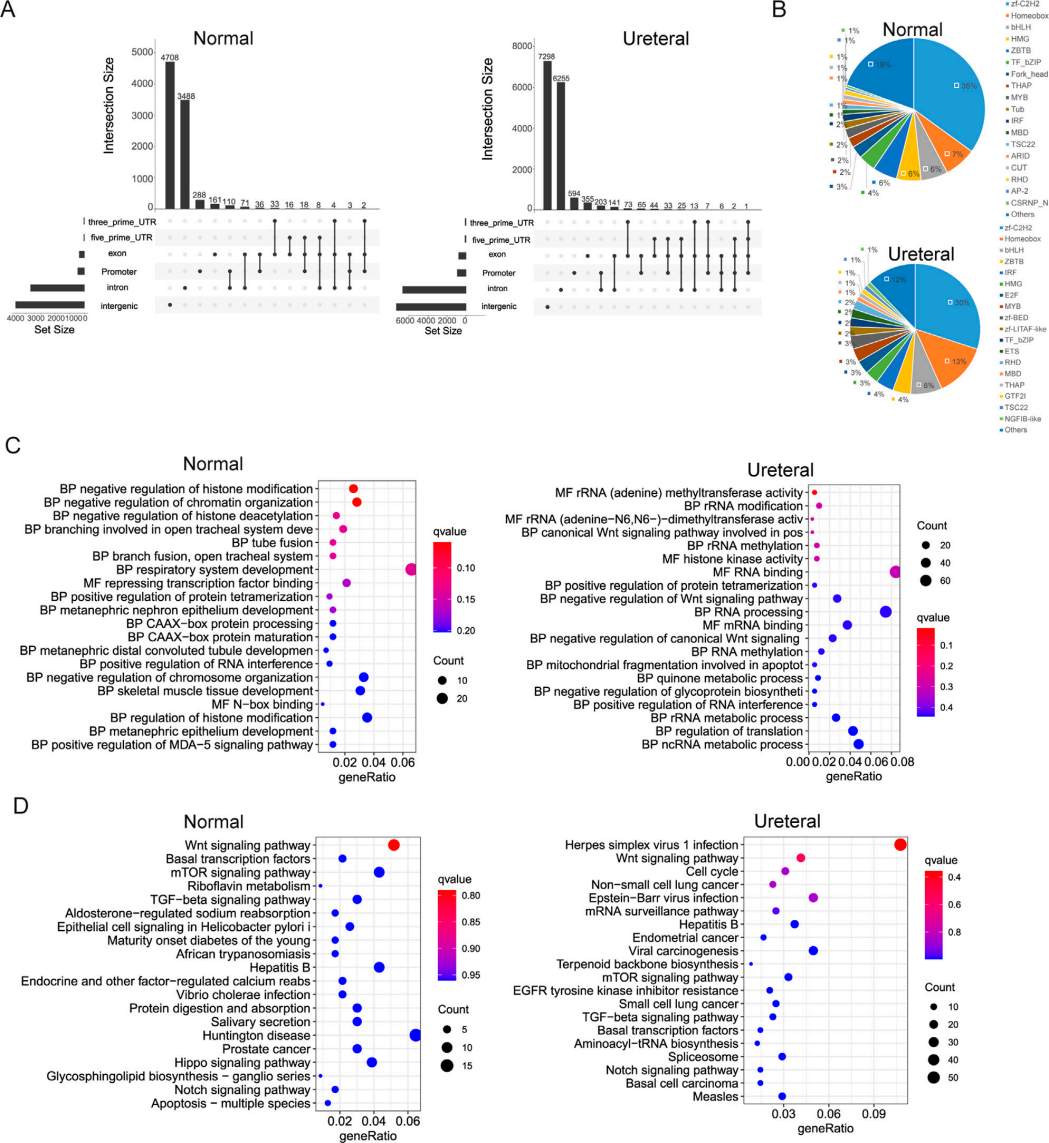
**(A)** Distribution of Normal (left) and Ureteral (right) open chromatin regions in genome. **(B)** Transcription factors identification of Normal (left) and Ureteral (right) peak target genes. **(C)** Go term enrichment of Normal (left) and Ureteral (right) peaks target genes. **(D)** KEGG pathway enrichment of Normal (left) and Ureteral (right) peaks target genes.

Generally speaking, cis-regulatory elements (cREs) will bound many transcription factors (TFs) to control the transcription level of genes. The binding of TFs requires exposure of DNA, which means chromatin states is openly. By ATAC-seq, we explored the chromatin open regions both in Normal and URETERAL, then detected differentially accessibly regions (DARs) between them to reveal different regulatory network between disease and normal kidney. Totally, we obtained 8,946 and 15,115 peaks in Normal and URETERAL, respectively. Distribution of ATAC peaks in genome is similar between Normal can URETERAL, that most of peaks located in intergenic regions (∼50%) or intron regions (∼40%), while only over 5% peaks located in promoter regions (Figure 3A).

We define the proximal gene as ATAC peaks target genes when gene TSS located within 5 kb of peaks. Then used iTAK (Zheng et al., 2016) to predict the transcription factors of peak related genes. Summary of TFs found in Normal open chromatin related genes includes zf-C2H2, Homeobox, bHLH, ZBTB, IRF, HMG, E2F, MYB, zf-BED, zf-LITAF-like, TF_bZIP, ETS, RHD, MBD, THAP, GTF2I, TSC22, NGFIB-like (Figure 3B). Exception of the TFs in Normal peaks related genes, other TFs, like Fork_head, THAP, Tub, CUT, ARID, AP-2, CSRNP_N also found in Ureteral ATAC peaks target genes. AP-2 family play role in kidney development. ZBTB, IRF and STAT are also potential factors regulate distal nephron fate (Muto et al., 2021). Among the STAT3 is to top candidate regulators (O'Brown et al., 2015). Fork_head family include FoxO1, which conformed to regulate E3 ligase then leads to muscle wasting in chronic kidney disease (Xu et al., 2012).

Further, Normal ATAC peaks target genes enriched at chromatin states related GO, such as histone modification, and chromatin organization (Figure 3C). As well as GO terms that related to tube or branch fusion and development, especially in nephron epithelium development. While the Ureteral open chromatin region targe genes enriched in Wnt signaling pathway, rRNA modification, RNA processing, apoptotic process, quinone metabolic process GO terms. Evidence revealed that Wnt signaling pathway controlling early nephrogenesis, and continuous activation result in renal fibrosis or kidney disease (Wang et al., 2018).

Both Normal and Ureteral ATAC peaks target genes KEGG pathway enriched in Wnt signaling pathway, mTOR signaling pathway, TGF-beta signaling pathway, Notch signaling, et al. (Figure 3D). Difference between Normal and Ureteral is that Normal enriched in Epithelial cell signaling and Hippo signaling, which is play important role in kidney development (Wong et al., 2016), while Ureteral enriched in EGFR tyrosine kinase inhibitor resistance. EGFR is the epidermal growth factor receptor, may overexpressed in many cancers, for example, non-small-cell lung cancer. And EGFR-TKIs now widely used in cancer treatment (Izzedine and Perazella, 2017). In kidney, EGFR activation may connect to TFG-β signaling, the inhibition of EGFR can release kidney damage and injury. Some EGFR-TKIs drugs been proved to improve kidney function, such as Gefitinib (Maruyama et al., 2015). In summary, the Ureteral accessible chromatin regions may affect the treatment of kidney disease through regulation of the EGFR-TKIs resistance genes.

Known motif enrichment in ATAC peaks by Homer showed that both Normal peaks and Ureteral peaks enriched SRS, TBP3, KLF family (KLF10, KLF5, KLF3), NRF,CRX, Trl, Sp1, NFγ, p63, Sp5, et al. (Supplementary Table S1). Highly similarity motif enrichment between Normal and Ureteral peaks may due to transcription factors highly enriched in overlapped peaks in two samples.

### Differentially accessible regions between normal and URETERAL

**FIGURE 4:**
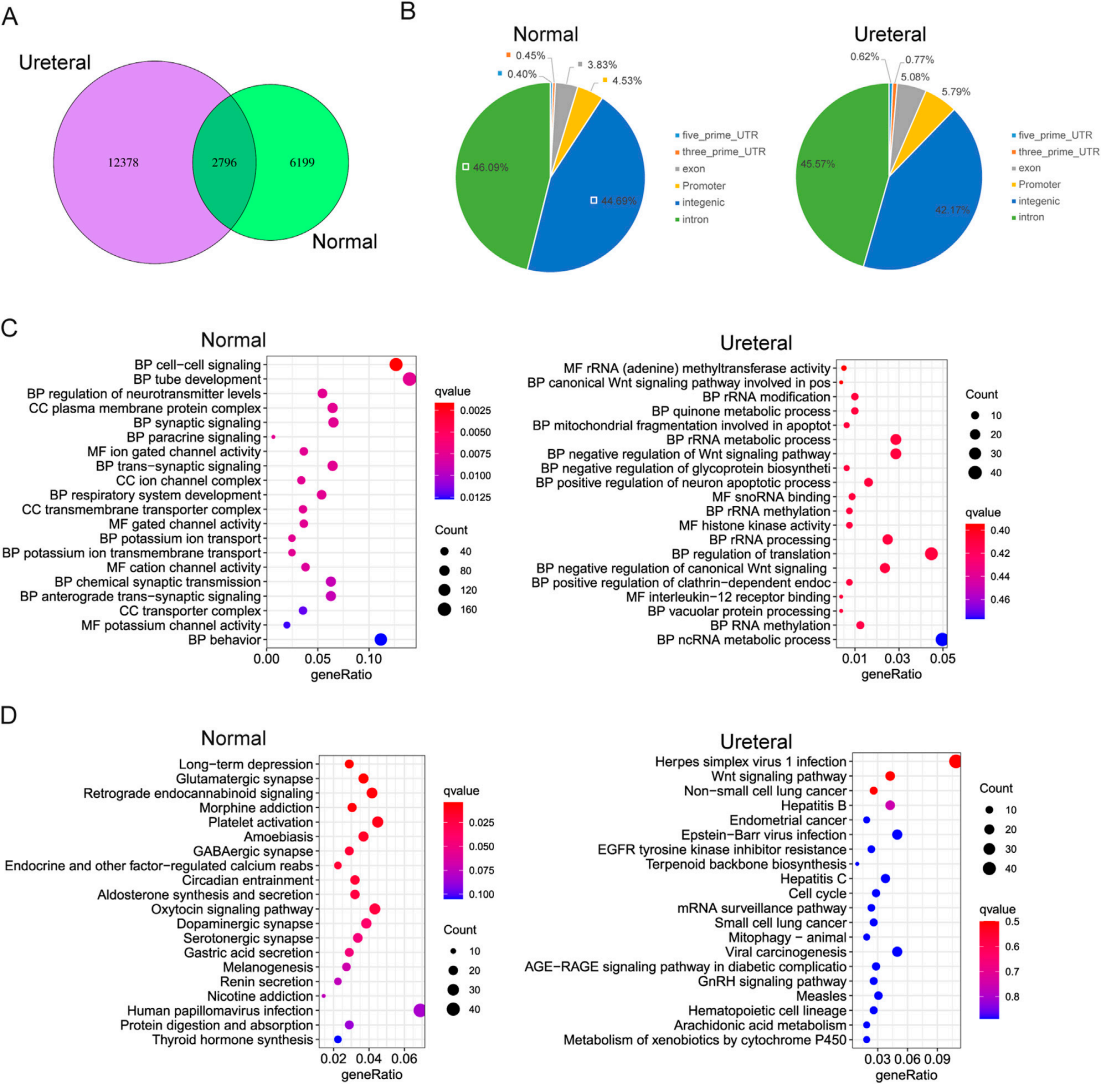
**(A)** Overlap of Normal and Ureteral peaks. **(B)** Distribution of Normal (left) and Ureteral (right) specific peaks in genome. **(C)** GO term enrichment of Normal specific (left) and Ureteral (right) specific DARs target genes. **(D)** KEGG pathway enrichment of Normal specific (left) and Ureteral (right) specific DARs target genes.

The chromatin states dramatically changed between Normal and URETERAL, that only small part of ATAC peaks exist both in Ureteral and Normal, while 12,399 and 6210 ATAC peaks specifically detected in Ureteral and Normal, respectively (FDR< 0.05, foldchange > 0, Figure 4A). Through chromatin open regions increased and extreme distinct in Ureteral compared to Normal, distribution of Normal specific DARs and Ureteral specific DARs are similar (Figure 4B). Most of ADRs located in intron and intergenic regions (over 45% and 40%), about 5% of DARs locate in promoter regions.

We also assigned the proximal genes within 5 kb as DARs target genes, then analysis GO enrichment of these DARs target genes. It showed that, Normal specific open chromatin regions target genes enriched in kidney related functions, such as cell-cell signaling, tube development, and paracrine signaling. Interestingly, they also enriched in ion channel or transport GO terms. While Ureteral specific DARs annotated genes related to Wnt signaling pathway, quinone metabolic process (Figure 4C). The latter one quinone may be toxic or protective to kidney, generally, reduced hemiquinones are free radicals that can damage DNA and cause cell death (Madeo et al., 2013).

KEGG enrichment of Normal DARs target genes seems related to neuron disease, or neuron functions. And these target genes also corelated to secretion, such as Oxytocin signaling pathway, Gastic acid secretion, Aldosterone synthesis and secretion. While in Ureteral DARs, their related genes enriched in kidney disease connected pathway, such as Wnt signaling pathway, Endometrial cancer and EGFR-TKI resistance (Figure 4D).

**FIGURE 5:**
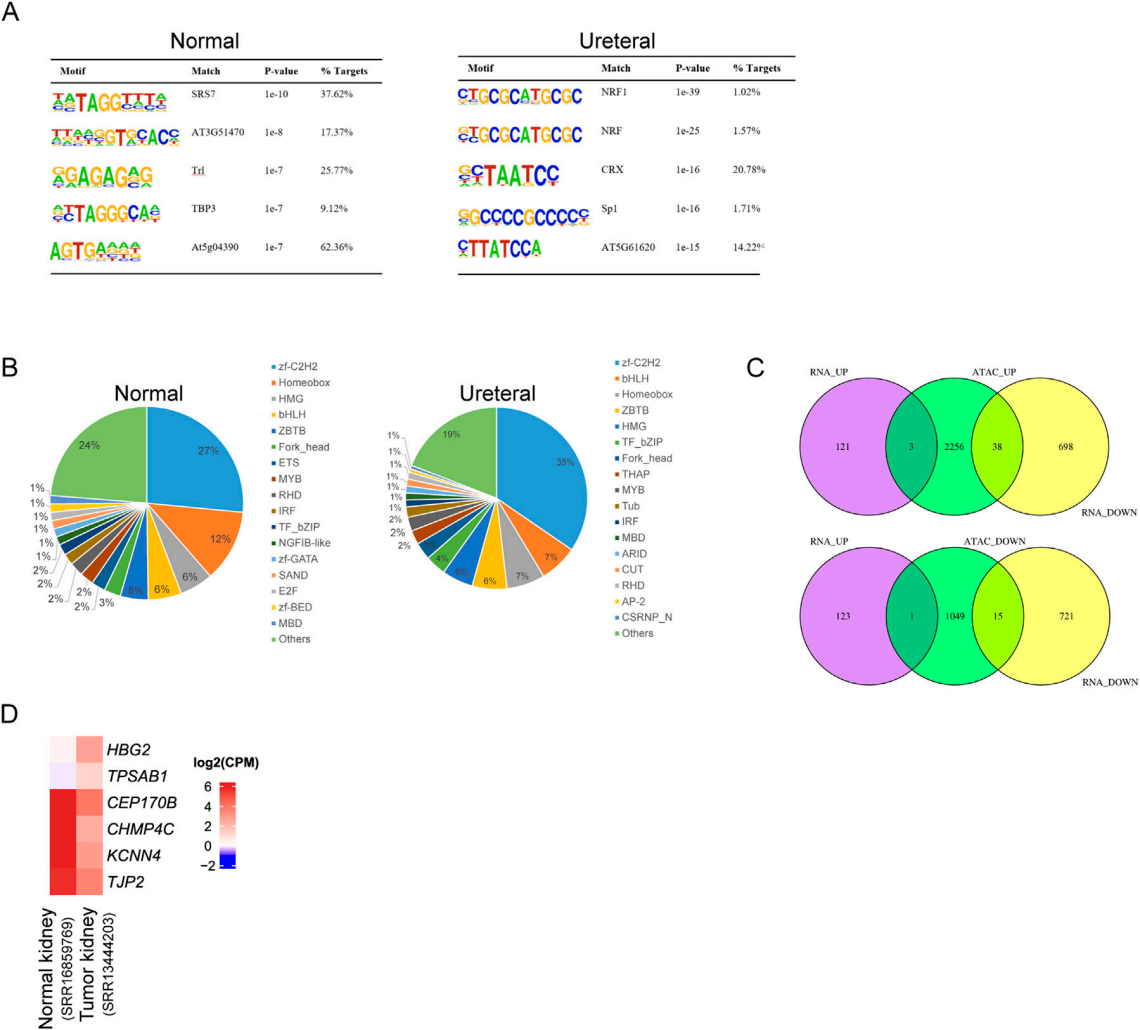
**(A)** Motif enrichment of Normal (left) and Ureteral (right) specific peaks. **(B)** Transcription factors identification of Normal (left) and Ureteral (right) specific peak target genes. **(C)** Venn diagram of ATAC peaks target genes and differential expressed genes. (Up) target genes of Ureteral specific peaks overlap with Normal up or downregulated genes. (Down) target genes of Normal specific peaks overlap with Normal up or down regulated genes.

Most TFs binding to motifs in chromatin open regions, differential accessible regions between Normal and Ureteral indicate potential disease specific TFs and regulatory networks. We used Homer to search known motifs in Normal and Ureteral specific ATAC peaks (Figure 5A), and found that SRS7, Trl, TBP3 enriched in Normal specific ATAC peaks, while NRF, CTX and Sp1 enriched in URETERAL. SP1 is a kind of C2H2 zinc finger factors, which can regulate apoptosis, fibrosis, and some pathological disorders. It's demonstrated that Sp1 can activate Klotho in renal tubular epithelial cells, and overexpression of SP1 will alleviate fibrosis in HK-2 by enhancer Klotho expression (Li et al., 2020), as well as alleviate acute kidney injury (Huang et al., 2021). It has reported that disease and health kidney are regulated by pathophysiological conditions of mitochondria (Duann and Lin, 2017). While NRF1 (nuclear respiratory factor) motifs, which usually activate genes for OXPHOS system and essential for mitochondrial biogenesis (Maldonado et al., 2021), is enriched in Ureteral ATAC peaks, that emphasize the importance of regulation of mitochondrial related genes in kidney disease.

Prediction of differential ATAC peaks target genes by iTAK showed the similar result to total ATAC peaks under each condition, except that Normal specific peaks target genes also predicted have zf-GATA, SAND (Figure 5B).

Though the chromatin states dramatically changed, the alternation of gene expression is inconsistency with chromatin states. Among Ureteral specific DARs related genes, only *HBG2*, *MYBL2*, and *TPSAB1* genes significantly upregulated in URETERAL, and 38 genes downregulated (Figure 5C). While, in Normal-specific accessible peaks, only TPSAB1 overexpressed in Ureteral and other 15 genes decreased in URETERAL. When focus on both open and actively expressed genes, *HBG2*, *MYBL2*, and *TPSAB1*. The former is hemoglobin gene, though it is not prognostic in renal cancer, the 5-year survival of low expression of *HBG2* is significantly higher than highly expressed ones. *MYBL2* is reported to play a role in many diseases, such as pan-cancer, hepatocellular carcinoma, renal caner. It can regulate synthesis of purine and promoter progression in HCC (Zhao JZ. et al., 2022). Furthermore, it is prognostic in renal cancer and pan-cancer, that high expression is unfavorable (Sun et al., 2020), (Chen et al., 2021). These results indicated that *MYBL2* is not only a potential target for HCC therapy but also have capacity in kidney disease cure.

Among 15 genes which downregulated in Ureteral sample, as well as the chromatin states closed in their potential regulatory sites, four genes (*CEP170B*, *CHMP4C*, *KCNN4*, and *TJP2*) are prognostic for renal cancer, that high expression is favorable. Two genes (*PAX8*, *UPK1A*) are highly expressed in kidney or urinary bladder. Other gene, like *HES1* is favorable for urothelial cancer. Together, these reduced genes confirmed that regulatory elements will change their chromatin states to alter their activity in controlling expression of genes importantly for maintaining normal function of kidney.

### Hydronephrosis modify the DNA methylation states barely

**FIGURE 6:**
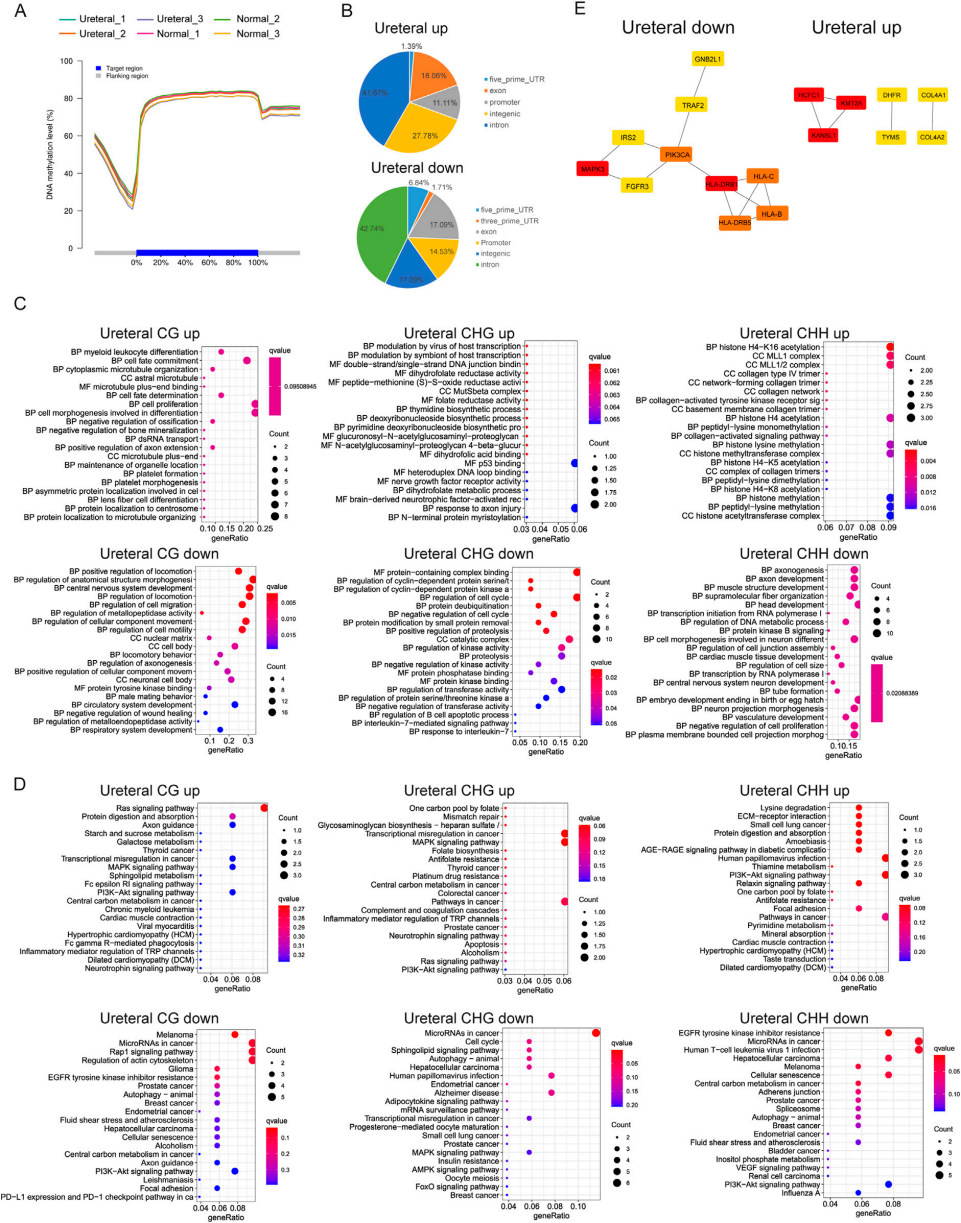
**(A)** DNA methylation level in different samples. **(B)** CG DMRs distribution in genome. CG, CHG, CHH DMRs GO term enrichment **(C)** and KEGG pathway **(D)**. **(E)** PPI network of Ureteral down DMRs (up, left) and Ureteral up DMRs (up, right) by STRING. And network of top hub genes identified by MCODE (down).

Methylation is one important modification in DNA that once DNA methylated, functional protein prevented to binding to DNA, then impacts the transcription of genes, generally silenced. It also reported that DNA methylation related to kidney function, such as methylation at gene *JAZF1*, *PELI1*, *CHD2*,*PHRF1*, *LDB2*, *CSRNP1* and *IRF5* (Schlosser et al., 2021). Many researches support that methylation alteration change *IRF5* expression in blood monocytes may affect immune pathway then influent kidney function (Graham et al., 2006). We used Bisulfite sequencing (BS-seq) to acquire genome-wide methylated sites. Methylation ratio of CG, CHG and CHH contexts in Normal is similar to that in URETERAL, about 98% CG methylated while only 7%–10% CHG or CHH methylated (Table 2). While the distribution of DNA methylation level around gene also similar between healthy and disease samples, as showed in Figure 6A, that methylation level is decreased dramatically proximal TSS, then recover to high level in gene body region. This makes sense that loading of transcription machine requires DNA exposure in transcription start site.

**TABLE 2:** Rate of DNA methylation in different context in Normal and Ureteral replicate samples.

Sample	Total_C	mC_rate	Total_CG	mCG_rate	Total_CHG	mCHG_rate	Total_CHH	mCHH_rate
URETERAL_1	378310170	10.34%	12605984	98.27%	76849691	7.39%	288854495	7.29%
URETERAL_2	451790056	10.25%	14995375	98.23%	91288158	7.29%	345506523	7.22%
URETERAL_3	841663950	14.01%	33648111	97.49%	176488036	10.56%	631527803	10.53%
Normal_1	373204632	10.16%	11716397	98.62%	74058151	7.31%	287430084	7.29%
Normal_2	305154004	10.25%	9352828	98.35%	59943399	7.47%	235857777	7.46%
Normal_3	630046784	12.29%	23096072	97.88%	130651383	8.99%	476299329	9.05%

DNA methylation varied between cell types, during development (Rakyan et al., 2008). Some studies demonstrated that differentially methylated regions (DMRs) were found between cancer and normal samples, and abnormal DNA methylation has been revealed in many diseases (Kim et al., 2011), (Hotta et al., 2018). Nevertheless, we explored only 152 CG DMRs (62 up, 90 down), 73 CHG DMRs (25 up, 48 down), and 102 CHH DMRs (40 up, 62 down) between Ureteral and Normal. This indicate that DNA methylation remain largely unchanged during hydronephrosis in genome widely.

DMRs distribution in genome exhibit that most of CG DMRs located in gene body, and over 10% of DMRs in promoter region in both Ureteral up or down DMRs (Figure 6B). Further analysis returned that up DMRs related genes (DMGs, within 2 kb upstream of TSS) in Ureteral sites enriched in myeloid leukocyte, cell fate, differentiation, proliferation, dihydrofolate activity, histone modification et al., most of enriched GO terms related to normal cell activity (Figure 6C). While the down DMGs enriched in regulation of locomotion, morphogenesis, also enriched in regulation of wound healing, circulatory system and tube formation. KEGG pathway of up DMGs (CG, CHG, CHH) deposited at PI3K-Akt signaling pathway, and CG, CHG also enriched at Ras, MAPK signaling pathway as well as inflammation related pathways (Figure 6D). MAPK signaling is a classical lipotoxicity pathway which display an important driving factor for loss of nephron in chronic kidney disease (Yuan et al., 2022). While both down CG and CHH DMGs enriched in EGFR tyrosine kinase inhibitor resistance and PI3K-Akt signaling pathway. The down CHH DMGs also enriched in bladder cancer, VEGF signaling pathway. Many research reported that downregulated nephrin may induced by inhibition of VEGF signaling and lead to many kidney diseases, such as nephritic syndrome or glomerular thrombotic microangiopthy (Izzedine et al., 2010). For CHG down DMGs, they deposited in Sphingolipid metabolites, MAPK, AMPK, FoxO signaling pathway. Among them, sphingolipid level is critical for maintaining renal function, abnormal expression of it may cause kidney disease (Mallela et al., 2022), and the suppressed AMPK also paly role in kidney injury through its function on renal tubular epithelial ion transport, while the FoxO signaling pathway may induce continuous autophagy in chronically hypoxic kidney through activation of Atg protein by accumulation of FoxO3 (Rajani et al., 2017).

We found that the DMRs target genes in Ureteral sample show similar to RNA-seq results that enriched in MAPK signaling pathway and PI3K-Akt signaling pathway, while it also shows common EGFR tyrosine kinase inhibitor resistance with ATAC-seq Ureteral specific peaks. These consistencies may reflect similarities in chromatin status and genes expression.

Even though current GO and KEGG analysis confirmed the importance of DNA methylation in regulation of kidney disease related genes, we still have limited acknowledgement about the regulatory network induced by DNA methylation. For better understand the regulate network, we analyzed co-methylation network of DMGs by STRING, then choose the top10 genes as hub genes (Figure 6E). Results display that *PIK3CA*, *MAPK3*, *HLA* family genes and critical in Ureteral down DMGs, and abnormal expression of them will influent kidney development or normal kidney function. While the *KMT2A*, *KANSL1*, *HCFC1* and *COL4A1* or *COL4A2*, *DHFR*, *TYMS* are hub genes in Ureteral up DMGs. Former three genes are transcription factor genes, participate general cell function, while the *COL4A1/2* usually related to many genetic disorders, such as kidney abnormalities, and mutation on *COL4A1* related to vesicoureteral reflux (VUR) (Kitzler et al., 2019).

To sum up, DNA methylation may not the main factor influence gene expression during hydronephrosis because of variation is not severe as chromatin states, but it still plays important role in kidney disease.

### Distal regulatory element modulates gene expression through 3D chromatin structure

Determination target genes of regulatory element is important to study abnormal gene expression in disease. Usually, we treat the proximal genes as their target genes, but this is not always the case, for we detected low overlap ratio between open chromatin regions proximal genes and true differentially expressed gene. Actually, when regulatory element and their target gene are far away apart in genome, they will form a transcription loop to start regulation.

To obtain the distal regulatory element and related genes, we can use 3D chromatin structure information from Hi-C or ChIA-PET experiments. In this study, we download the public Hi-C data of kidney from ENCODE, and obtained over 4,500 loops in 10 kb resolution and 3,700 topologically associated domains.

**FIGURE 7:**
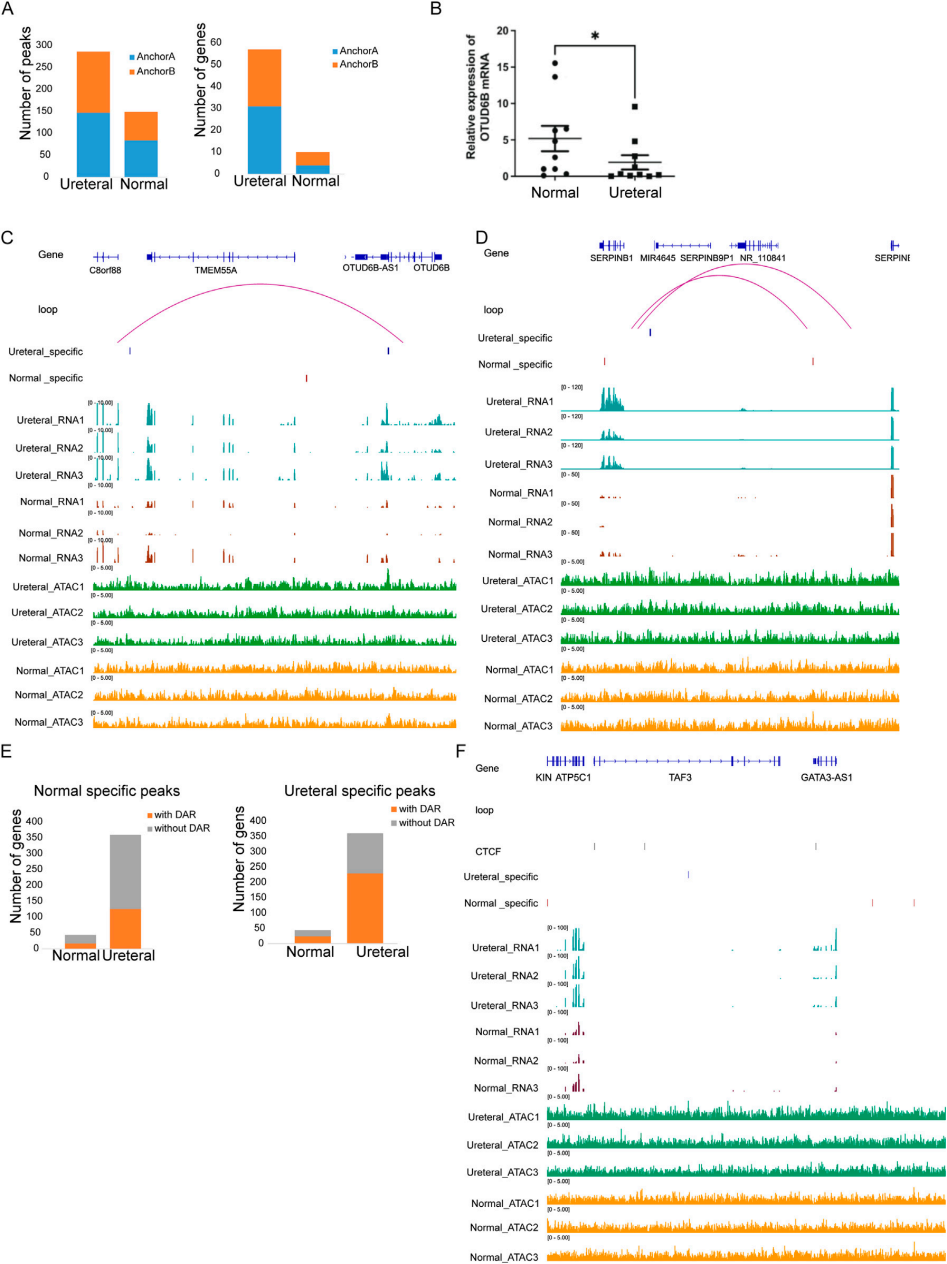
**(A)** Numbers of DARs (left) and DEGs (right) in loop anchor (anchor A, anchor B) between Ureteral and Normal samples. Visualization of chromatin loop between gene *OTUD6B* **(B)** and *SERPINB1* **(C)**. **(D)** Number of Ureteral upregulated genes with or without Normal (left) and Ureteral (right) specific peaks in same TAD. **(E)** Visualization of chromatin structure and states near *GATA3* genes.

Then we try to search loop that one anchor to DAR another anchor to promoter of DEG (-TSS up 1 kb to down 500 bp) between Ureteral and Normal. Impressively, Through, hundreds of DARs (147 + 139 of Ureteral specific peaks, 84 + 65 of Normal specific peaks) and dozens of genes (4 + 3 of Normal upregulated genes, 31 + 26 Ureteral upregulated genes) located in anchors of loop (Figure 7A), only two DEGs (*OTUD6B* and *SERPINB1*) formed loop with DARs.

*OTUD6B* is a new identified ovarian-tumor (OUT) deubiquitylating enzyme families, and a biomarker in most cancer. It has reported that *OTUD6B* downregulated in kidney clear cell renal cell carcinoma, kidney renal papillary cell carcinoma compared to healthy sample (Zhao G. et al., 2022), (Guo et al., 2022), and its expression level correlated with survival in VHL missense mutation patients. In this experiment, we also found that the expression of OTUD6B in the XZ site was significantly decreased compared with the normal site through RT-PCR results. High level of *OTUD6B* in Ureteral sample compared to Normal and clear loop between Ureteral specific peaks (∼100 kb upstream of TSS) and *OTUD6B* promoter region (TSS upstream 1 kb to downstream 500 bp) is detected (Figure 7B). This result confirmed that distal regulatory element control expression of kidney disease related genes through loop.

*SERPINB1* is serpin family B member 1, which could protect tissues from injury at inflammatory regions. Their family member SERPINB2 also associated with renal injury and kidney disease through regulation of macrophage phagocytosis and migration (Sen et al., 2020). Unlike the open chromatin state facilitate the gene expression of *OTUD6B* in Ureteral sample, *SERPINB1* promoter formed a loop with Normal specific peak while the expression level is low in Normal (Figure 7C). This result may be attribute to the opposite correlation between chromatin states and gene expression level, that the activation of SERPINB1 gene in Normal call for condensed chromatin.

TAD is a small structure that confine affect range of regulatory element, prevent their activity on neighbor TAD. Though the regulatory pairs between RE and target genes detected in our study is low, we found that TAD structure have impressively impact on transcriptional regulation of gene. About half DEGs within TAD (44 Normal increased genes, and 360 Normal depressed genes), and nearly 50% of them coupled with DARs in same TAD (Figure 7D), higher ratio than DEG genes overlap with DAR related genes (Figure 5C). And over half of Ureteral increase genes located TAD contain Ureteral specific peaks (Figure 7D, right), higher than Ureteral highly expressed genes located TAD contain Normal specific peaks (Figure 7D, left).

For example, *GATA3* which involved in Volffian duct formation, once inactivated will result in complete deficiency of reproductive tract, ureter, and kidney, is highly expressed in Ureteral compared in Normal (adj.p < 0.05). And the ATAC-seq data showed that many cREs located within the same TAD of *GATA3*, include four Ureteral REs, and seven Normal REs. The remarkable thing is that proximal Ureteral REs located far away from promoters (125 kb), far than proximal Normal REs (68 kb). The possible reason is that there are two CTCF binding sites located near Ureteral REs and promoter of *GATA3*, which may induce the transcription loop between Ureteral REs and *GATA3* (Figure 7E). Nevertheless, the detail mechanism of Ureteral RE regulate *GATA3* may need further investigate.

Taken together, these results showed the chromatin structure, both chromatin loop and TAD have limiting effects on regulate activity of cREs.

## Conclusion

Hydronephrosis is a disease that disturbs people’s health, increase the risk of developing chronic kidney disease. For better understanding the regulatory network of hub genes in pathogenesis of hydronephrosis, we compared transcriptome and epigenetic states between ureteral stricture segment and normal segment in patient.

Generally, there were 127 genes overexpressed in normal ureter, and these genes were enriched in inflammatory response, which means dysfunctional immune in hydronephrosis. While 736 decreased gene in ureteral stricture were related to kidney disease correlated biology process or pathway, such as *PLA2G2F* which highly expressed in damaged kidney and receptor of PLA2 demonstrated to be marker of nephropathy disease, as well as PPAR, Hippo and PI3K-Akt signaling pathway, which have been reported affect normal development of kidney and lead to renal disease.

Along with further investigation, we also found many hub genes associated with upregulated genes in hydronephrosis samples. For example, *BCL6*, which inhibits NF-k β signaling and may inhibit renal inflammation (Chen et al., 2017), is negatively correlated with GFR. And all upregulated hub genes enriched in urinary system disease. This provides us new thought in exploring new treatments for hydronephrosis treatment.

Alteration of chromatin states between Normal and Ureteral samples figure out that, chromatin accessibility would affect disease related genes. As showed in our study, genes targeted by disease tissue specific open chromatin regions enriched in EGFR inhibitor resistance, which reported that EGFR tyrosine kinase inhibitor can release kidney injury (Izzedine and Perazella, 2017; Maruyama et al., 2015). Means the disease will overexpress the EGFR-TKIs resistance related genes to perturb damage repair. Following exploration on TF binding motifs showed that NRF, which may enhancer OXPHOS system related genes are essential for mitochondrial biogenesis, is enriched in Ureteral ATAC peaks, that emphasize the importance of regulation of mitochondrial related genes in kidney disease.

Transcription level not always consistent with alteration of chromatin states. In this study, we found only 3 and 15 over expressed genes in disease and normal sample, respectively, consistent with their chromatin accessibility under two conditions. Interestingly, *MYBL2*, which control the synthesis of purine and important in many diseases, is highly expressed in disease tissue and prognostic in renal caner (Sun et al., 2020; Chen et al., 2021). These results indicates that *MYBL2* may be potential treatment target.

DNA methylation is also an important regulation method in gene transcription. Though our result showed little overlap between DMGs and DEGs of mRNA, the GO and KEGG enrichment analysis of DMGs still evidenced the relationship between DNA methylation states and reported signaling pathway, such as MAPK and FoxO (Mallela et al., 2022; Rajani et al., 2017), in kidney injury on the other side.

We also explored the function of chromatin structure in mediating transcriptional regulation during hydronephrosis, and found an interaction between Ureteral specific open chromatin regions and promoter of *OTUD6B* gene, which is a kidney disease related gene (Zhao G. et al., 2022; Guo et al., 2022) and highly expressed in disease tissue compared to normal tissue. This illustrated the importance of chromatin structure in disease process. On the other hand, this study showed that TAD also affect the expression level by restricting the regulation range between regulatory element and their target genes.

In summary, we found many hub genes and potential therapeutic target during hydronephrosis, and also confirmed that epigenetic play important role in gene expression and relevant in disease progress.

Renal tubule epithelial cells and vascular networks are severely damaged, interstitial fibrosis and inflammatory cells are rapidly infiltrated are the classical states in hydronephrosis. Understanding the interactions between different cell death pathways is important, especially to target these pathways for therapeutic purposes. Our findings reveal an important link between tubular cell pyrosis and obstructive nephropathy.

## Materials and methods

### Sampling

The inclusion criteria included patients MUO caused by an abdominal or pelvic malignancy with overt hydronephrosis that was documented on ultrasonography (US) or computed tomography (CT) scans. Sample collection method: After the patient undergoes the relevant surgery, professional medical staff collect samples in a strictly sterile environment. First, the surgical area is meticulously cleaned to ensure that the surrounding environment is free of any contaminants that may compromise the sample. For the tissue at the ureteropelvic junction, the medical staff carefully manipulated the distal wire, accurately identifying and selecting distal normal ureteral tissue that was more than 2 cm away from the stenotic segment. Using strictly sterilized surgical instruments, approximately 0.5 g of tissue was precisely transversely cut to serve as the experimental group (ZC group, n = 10). Subsequently, under sterile conditions, the proximal stenotic segment of the ureter specimen was selected, and 0.5 g was transected in the same manner for the control group (XZ group, n = 10). Throughout the entire collection process, medical staff moved gently and accurately to minimize unnecessary damage to the tissue, while consistently maintaining the integrity and purity of the samples. After repeated washing with physiological saline under sterile conditions, the samples were immediately stored in liquid nitrogen and subsequently transferred to a −80°C refrigerator for later use. Ethical approval status: The research protocol was approved by the Ethical Committee of the Henan Provincial People’s Hospital (Zhengzhou, Henan, China 2322122014).

### RNA-seq

Total RNA extracted from ureteral stricture segment (URETERAL) and normal segment (Normal) sample using the RNAprep Pure Kit DP432 (TIANGEN Biotech Co., Ltd., Beijing, China). Each with three replicates. After extraction of RNA, we used VAHTS mRNA-seq V3 Library Prep Kit and 1 μg of total RNA to construct the illumine sequencing library. Briefly, we firstly collect RNA by oligo (dT) beads, then fragment RNA and reverse them by random hexamer primer. Finally, RNA was sequenced by Illumina Novaseq 6000 in 150 nt paired-end model. Quality control was assessed by Qsep400 instrument.

### ATAC-seq

Three replicate samples from ureteral stricture segment (URETERAL) and normal segment (Normal) were taken, respectively. Approximately 0.1 g samples were collected and immediately grinded in 2 mL of pre-chilled lysis buffer (15 mM Tris-HCl pH 7.5, 20 mM NaCl, 80 mM KCl, 0.5 mM permine, 5 mM 2-ME, 0.2% TritonX-100). The total mixture was filtered with miracloth then loaded on the surface of 2 mL dense sucrose buffer (20 mM Tris-HCl Ph 8.0, 2 mM MgCl_2_, 2 mM EDTAl, 15 mM 2-ME, 1.7 M sucrose, 0.2% TritonX-100) in a 10 mL Falcon tube after grinding. The nuclei were centrifuged at 2,200 g at 4C for 15 min and the pellets were resuspended in 500 ul pre-chilled lysis buffer.

Mix 25 ul of reaction buffer, 2.5 ul of Nextera Tn5 Transposase, and 22.5 ul of Nuclease free H_2_O to make the transposition reaction mix. Crude nuclei was resuspend in the transposition reaction mix and incubated at 37°C for 30 min. Using a Qiagen MinElute PCR Purification Kit to purify the DNA after transposition. The DNA was amplified using NEBNext High-Fidelity 2x PCR Master Mix for 10–15 cycles. Amplified libraries were purified by using Qiagen MinElute PCR Purification Kit. The library was eluted in 20 μL Elution Buffer (10mM Tris Buffer, pH 8). The quality of purified libraries was assessed using Bioanalyzer and Q-bit, then sequenced using Illumina novaseq 6000 with PE 150 method.

### DNA methylation

Whole Genome Bisulfate Sequencing were performed on ureteral stricture segment (Ureteral) and normal segment (Normal) samples, each with three replicates. Totally, 1ug DNA extracted from samples, and fragmented with an ultrasonic disruptor (Bioruptor), in average 300–500 bp size. In order to evaluate bisulfite conversion rates, 20 ng nonmethylated lambda DNA (251431, Promega) were spiked in as controls during library preparation. Fragmented DNA were end-repaired and ligated to a fully methylated adapter by using NEXTflex™ Bisulfite-Seq Barcodes-6 Kit (51191, Bioo Scientific). EZ DNA Methylation-Gold™ kit (D5005, Zymo Research Corp) was used for bisulfite conversion of adapter-linked DNA. After PCR amplification, around 300 bp DNA fragments were used for sequencing under Illumina platform.

### RNA-seq analysis

The adapter and low-quality reads were removed by cutadapt (version 1.11) (Martin, 2011). Clean reads were mapped to the human hg38 reference genomes by Hisat2 (version 2.1.0) (Kim D. et al., 2015), allowing up to two mismatches. These genes were subjected to alignment against public protein databases; NR (RefSeq non-redundant proteins). Featurecount (v1.6.0) (Liao et al., 2014) was used for transcript abundance estimation and normalization of expression values as FPKM (Fragments per kilobase of transcript per million fragments mapped). Differentially expressed genes were identified by edgeR (Chen et al., 2016) with FDR<0.05 and |log2FoldChange| > 1. Briefly, we removed low count genes with filterByExpr function, and normalized the read counts with TMM method by calcNormFactors function. Then, we used negative binomial (NB) model to estimate the dispersion parameter by estimateDisp function and generalized linear model (GLM) to fit the dispersion. Finally, exactTest function were used to detect differential expressed genes and Beniamini and Hochberg’s algorithm were use to correct the p value.

### ATAC-seq analysis

The adapter and low-quality reads were removes by Trimmomatic (version 0.36) (Bolger et al., 2014). Clean reads were mapped to the human hg38 genome by Hisat2 (version 2.1.0), allowing up to two mismatches. PCR duplicated were removed by Samtools (version 1.3.1) (Li et al., 2009). Then uniquely mapped reads were call peaks using MACS2 software (version 2.1.1.20160309) (Zhang et al., 2008) by default parameters (nomodel; shift −100; extsize 200; model fold, 5, 50; q value, 0.05). The differentially accessibly regions were identified by DESeq2 method according to Diffbind, with FDR <0.05 and |log2FoldChange| > 0 under default parameter (version 1.16.3) (Ross-Innes et al., 2012). If the midpoint of a peak located closest within 5000bp around TSS of one gene, then this gene will be assigned as the target gene. HOMER (version3) (Heinz et al., 2010) was used to predict motif occurrence within peaks with default settings for a maximum motif length of 12 base pairs.

### DNA methylation analysis

Trimmomatic (version 0.38) was used to filter out low-quality reads. The clean reads were then mapped to reference genome hg38 by the bitmapperBS (Cheng and Xu, 2018). Bisulfite conversion rates was evaluated by lambda DNA. The coverage of Cytosine bases across the genome and Cytosine-base methylation levels in various types were counted by cgmaptools (Guo et al., 2018). Methylation level was determined by dividing the number of reads covering each mC by the total number of reads covering that cytosine, which was also equal to the mC/C ratio at each reference cytosine. Circlize (Gu et al., 2014) was used to do the whole genome methylation distribution analysis. DNA methylation regions (DMRs) were calculated by gmaptools (Song et al., 2009). DMR-associated genes were defined as genes with the closet DMR located within 2 KB upstream of the transcription start site and 2 KB downstream of the transcription end site.

### GO and KEGG analysis

ClusterProfiler was employed to perform GO and KEGG enrichment analysis with a q value cutoff of 0.05.

### Real-time PCR (RT-PCR)

The mRNA levels were assessed by reverse transcription PCR (RT-PCR). RT-PCR was performed as previously reported and was assessed on a ABI 7900HT Real-time PCR system (Applied Biosystems, Foster city, CA, United States) using SYBR premix Ex Taq TM (Takara Bio, Kusatsu, Shiga, Japan). The relative quantities of target genes were calculated from triplicate samples after normalization by an internal control. RT-PCR was performed as measured for 35 cycles at 94°C for 20 s, 58°C for 30 s, and 72°C for 45 s. Oligonucleotide primers are indicated in Table 3.

**TABLE 3:** Primer sequences used for RT-PCR.

H-PLA2-F	AAG​GAA​GCC​GCA​CTC​AGT​TA
H-PLA2-R	TTG​CAC​AGG​TGA​TTC​TGC​TC
H-OTUD6B-F	GCG​AGA​AGA​ACG​GAT​AGC​TG
H-OTUD6B-R	TCT​CAA​GGC​AAC​CAC​AGT​CA
H-HBG2-F	GCA​AGA​AGG​TGC​TGA​CTT​CC
H-HBG2-R	GAA​TTC​TTT​GCC​GAA​ATG​GA
H-MYBL2-F	AGA​AAC​GAG​CCT​GCC​TTA​CA
H-MYBL2-R	AGA​TGG​TTC​CTC​AGG​GAG​GT
H-TPSAB1-F	CAG​TTC​TAC​ACC​GCC​CAG​AT
H-TPSAB1-R	CGC​TCA​TCA​TTG​TCC​ACA​TC
H-β-Actin-F	TGG​ACT​TCG​AGC​AAG​AGA​TG
H-β-Actin-R	GAA​GGA​AGG​CTG​GAA​GAG​TG

## Data Availability

The data presented in the study are deposited in the Genome Sequence Archive repository, accession number GSA: HRA005137, https://ngdc.cncb.ac.cn/gsa-human/browse/HRA005137.
